# Effectiveness of worksite wellness programs based on physical activity to improve workers’ health and productivity: a systematic review

**DOI:** 10.1186/s13643-023-02258-6

**Published:** 2023-05-24

**Authors:** Maria Marin-Farrona, Brad Wipfli, Saurabh S. Thosar, Enrique Colino, Jorge Garcia-Unanue, Leonor Gallardo, Jose Luis Felipe, Jorge López-Fernández

**Affiliations:** 1 IGOID Research Group, Department of Physical Activity and Sport Sciences, University of Castilla-La Mancha, 45071 Toledo, Spain; 2 OHSU-PSU School of Public Health, Portland State University, Portland, OR 97201 USA; 3 Oregon Institute of Occupational Health Sciences, Oregon Health & Science University, Portland, OR 97239 USA; 4 Exercise and Sport Science, Faculty of Health Sciences, Universidad Francisco de Vitoria, Ctra. Pozuelo-Majadahonda Km 1,800, Pozuelo de Alarcón, 28223 Madrid, Spain; 5 Faculty of Sport Sciences, Universidad Europea de Madrid, Calle Tajo S/N, Villaviciosa de Odón, 28670 Madrid, Spain

**Keywords:** Work-ability, Exercise, Cardiorespiratory, Employee, Company, Occupational health and safety

## Abstract

**Background:**

Although the scientific literature has previously described the impact of worksite programs based on physical activity (WPPAs) on employees’ productivity and health in different contexts, the effect of these programs has not been analyzed based on the characteristics or modalities of physical activity (PA) performed (e.g., aerobic exercise, strength training, flexibility). In addition, studies on WPPAs usually report health and productivity outcomes separately, not integrated into a single study. Knowing the health and economic-related impacts of a WPPAs could provide useful information for stakeholders and policy development.

**Objective:**

The purpose of this review was as follows: (1) to analyze the effect of different modalities of WPPAs on employees’ productivity and health and (2) to investigate the economic impact of WPPAs.

**Methods:**

This systematic review is registered in PROSPERO (CRD42021230626) and complies with PRISMA guidelines. Only randomized controlled trials from 1997 to March 2021 were included. Two reviewers independently screened abstracts and full texts for study eligibility, extracted the data, and performed a quality assessment using the Cochrane Collaboration Risk-of-Bias Tool for randomized trials. Population, instruments, comparison, and outcome (PICO) elements were used to define eligibility criteria. Eight-hundred sixty relevant studies were found through electronic searches in PubMed, Web of Science, Medline, Scopus, and SPORTDiscus databases. Once the eligibility criteria were applied, a total of 16 papers were included.

**Results:**

Workability was the productivity variable most positively impacted by WPPAs. Cardiorespiratory fitness, muscle strength, and musculoskeletal symptoms health variables improved in all the studies included. It was not possible to fully examine the effectiveness of each exercise modality because of the heterogeneity in methodology, duration, and working population. Finally, cost-effectiveness could not be analyzed because this information was not reported in most studies.

**Conclusion:**

All types of WPPAs analyzed improved workers’ productivity and health. However, the heterogeneity of WPPAs does not allow to identify which modality is more effective.

**Supplementary Information:**

The online version contains supplementary material available at 10.1186/s13643-023-02258-6.

## Background

Traditionally, worksite wellness programs (WWPs) have mainly focused on individually based initiatives around nutrition, physical activity (PA), and smoking [1]. However, more integrated approaches that consider environmental and structural level factors leadership, health, psychosocial factors, and safety, such as the *Total Worker Health*® approach, are becoming more accepted by organizations [1, 2]. Therefore, recent WWPs focused on promoting employee health include different tactics (e.g., health assessments, education, counseling) that target parameters such as PA levels (quantity of light, moderate, or vigorous activity), stress levels, or weight control to improve employees’ health and productivity [3].

One of the main reasons for the growing number of WWPs is to reduce employee absenteeism as a consequence of the increased burden of musculoskeletal disorders [4] and chronic diseases such as lung disease [5], metabolic syndrome [6], burnout syndrome [7], cardiovascular diseases [8], and obesity [9]. Modifiable health risk factors (e.g., smoking, poor nutrition, physical inactivity) are a significant factor in developing these diseases and are therefore targets of WPPs [1]. Among all of these parameters, the promotion of PA at the workplace is increasing in interest because it might effectively reduce the risk of chronic disease while improving productivity and reducing healthcare costs [10]. Accordingly, the World Health Organization encourages the promotion of PA at the workplace within the Global Action Plan on PA (Action 2.5; Action 3.3), while the promotion of health and PA at the workplace is also included among the Sustainable Development Goals (goals 3 and 8) [11]. As a result, it is increasingly common to find organizations with worksite programs based on PA (WPPAs) [12]. These programs can be tailored to factors like the targeted population (e.g., office workers, builders, doctors), the PA structure (i.e., unstructured, semi-structured, or structured), the social setting of PA (i.e., alone or in a group), the use of behavior change strategies [13], and, according to the WHO, the type of PA modality such as aerobic exercise (AE), strength exercise modality (ST), and flexibility or balance exercise (FL) [11].

The impact of WPPAs is often reported in terms of productivity (i.e., work-ability, absenteeism, job satisfaction) or health (i.e., cardiorespiratory fitness), with promising results [14]. For example, WPPAs have been effective in improving variables like cardiorespiratory fitness and presentism, resulting in a financial return and lesser healthcare costs, among others [14]. Therefore, contemporary workplaces may benefit from the implementation of WPPAs in several ways. However, despite several reviews on WPPAs [12, 15–17], none of them has analyzed the impact of these programs on both productivity and employees’ health at the same time through randomized controlled trials (RCTs). Also, it remains unknown which type of PA modality included in WPPAs (i.e., AE, ST, FL, or a combination of AE and ST [CO]) is more effective for improving both productivity and health. Finally, understanding the economic impact of WPPAs would provide useful information in developing preventive proposals in companies and encourage workers to adopt more active lifestyles.

Therefore, the purpose of this review was to (1) to analyze the effect of WPPAs by PA modality (AE, ST, FL, and CO) on both employees’ productivity and health and (2) to investigate the cost-effectiveness of WPPAs targeting both productivity and workers’ health.

## Methods

This systematic review was carried out and reported following the Preferred Reporting Items for Systematic Reviews and Meta-Analyses (PRISMA) guidelines [18]. The protocol was preregistered in the PROSPERO database with registration number CRD42021230626.

### Study eligibility

Only RCTs published in English, delivered at a company, which included any form of PA either at work or in leisure time were included. No restrictions were applied regarding whether the WPPAs were implemented by a research team or researchers in collaboration with the organization. Population, intervention, comparison, and outcome (PICO) elements were used to define eligibility criteria (Table 1). Studies had to be performed from 1997 as this was the year of the inception of the European Network for Workplace Health Promotion to March 2021. Studies only focusing on workplace safety and accident prevention, reviews, methodological papers, case–control, cohort, observational studies, and conference proceedings were excluded. For inclusion, articles needed to report pre- and post-test results either for intervention or control groups and had to report both productivity and health outcomes. Finally, interventions where physical activity was embedded in a broader program were included.

**Table 1:** Eligibility criteria for studies: definition of the PICO elements

PICO elements	Inclusion criteria
Population	No gender, age, type of industry, or country restriction was applied. Workers on sick leave or non-working populations were excluded. Only those programs designed for the nonclinical population were accepted. Participants had to work for the company delivering the WPPAs^b^ program during the time the intervention was delivered and had to participate actively in the intervention
Intervention	Programs had to include any form of PA either at work or in leisure time (e.g., lunch walks, fitness centers, exercise groups). PA^c^ could be delivered offline, online, or as a combination of both
Comparison	Employees of an organization providing no WPPAs. Other interventions not including PA like ergonomics or health promotion activities
Outcomes	(1) Primary outcome: Productivity and performance (measured through self-reported health-related productivity questionnaires and/or objective measures (e.g., typing speed and accuracy, mouse proficiency) will be considered. Absenteeism (using workers’ sick leave) and presenteeism (quantitative [e.g., number of calls made by an employee] and qualitative [e.g., questionnaires]) (2) Secondary outcome: Health variables (measured as “natural units” (e.g., MET minutes, energy expenditure, time of moderate/vigorous PA, sitting/standing time, body mass index (BMI)/overweight etc.) or as proportions (e.g., number meeting the PA guidelines). Smoking, glucose levels, cholesterol levels, sleep patterns, pain, quality of life, mental health or health risks and diet, self-reported hours sitting per workday, stress, anxiety, and other health outcomes (3) Tertiary outcome: Economic benefits/impact (when reported) were analyzed (ROI^a^, medical claims, cost of absenteeism, cost of presenteeism, staff turnover, etc.)

^a^*ROI*, return of investment

^b^*WPPAs*, worksite wellness programs based on physical activity

^c^*PA*, physical activity

### Information sources and search strategy

Between January and March 2021, a comprehensive and systematic literature search was performed through five databases in health and physical activity, as well as organizational sciences: PubMed, Web of Science, Medline, Scopus, and SPORTDiscus. The following key terms were combined with “AND” or “OR” and searched on each database: (1) workplace, (2) worksite health promotion program, (3) employee, (4) worker, (5) productivity, (6) PA, (7) exercise (8) health, (9) cost-effectiveness, and (10) cost–benefit. Also, reference lists of identified and relevant reviews were screened. Additionally, we conducted a keyword search in Google Scholar and a backward and forward tracking. Automatic notifications from database searches were set, and relevant studies were continuously added until March 16, 2021. The full search strategies for all databases can be find on Supplementary Table S1.

### Study selection process

Literature searches and inclusion/exclusion decisions were completed by two independent researchers. Search results were stored in reference manager software Mendeley Desktop v 1.17.13 (Mendeley Ltd., London, UK). After removing duplicates, titles and abstracts were screened. Full texts of relevant studies were consulted for definitive inclusion. A consensus discussion between the researchers took place after title and abstract screening and again after full-text consultation.

### Data collection process

A form was used to obtain the follow information: (1) article details (reference, affiliation, publication year, country, study design); (2) characteristics of study participants (setting type of industry, average age, sex distribution, and sample size); (3) details of the intervention (focus of intervention, description of intervention, duration, control, and intervention group characteristics); (4) outcomes (productivity, health, and economic evaluation); and (5) conclusion of the study. In order to analyze the effect of different PA modalities on the selected outcomes, the type of intervention was grouped into four different modalities: (a) aerobic exercise (AE), for example, running, cycling, walking, rowing, Nordic walking, and dancing; (b) strength training (ST), for example, with dumbbells and barbells, gym programs, isometric exercises, elastic bands; (c) flexibility or balance exercise (FL); or (d) combined aerobic and strength exercise (CO) such as high-intensity interval training or similar activities.

### Risk of bias in individual studies

The data extraction, quality assessment, and determination of the risk of bias were performed independently and in duplicate by two investigators, using the Cochrane Collaboration Risk-of-Bias Tool for randomized trials [19]. Discrepancies were solved by a discussion leading to consensus or through consultation with a third reviewer (J. F.) in accordance with the Cochrane Collaboration guidelines. Items included were as follows: random sequence generation (selection bias), allocation concealment (selection bias), blinding of participants and personnel (performance bias), blinding of outcome assessment (detection bias), incomplete outcome data (attrition bias), and selective reporting (reporting bias). The maximum score is 100% (low scores indicate a higher risk of bias).

### Outcomes and data synthesis

Two independent researchers (M. M. F. and J. L.) extracted data on study characteristics and outcomes of productivity, health, or economics evaluations and captured these data in prepared digital forms. For each measurement of interest, we recorded the sample size, mean, standard deviation, and *p*-value. We then calculated the effect size with a post hoc standardized mean differences (Cohen’s d) using G*Power 3.1 [20].

The Supplementary Table S2 provides details on the main outcome, while information on the characteristics and outcomes of the studies analyzed, such as productivity, health, and economic variables, can be found in the description and inclusion criteria of the studies. The effect size of workplace physical activity interventions on these outcomes is displayed in Supplementary Table S3. The collected information based on the type of variable (productivity and health) and the intervention modality (A. E., S. T., F. L., and C. O.) was categorized to synthesize the data in Table 2. Additionally, symbols + , -, and = were used to represent positive, negative, and neutral effect of the intervention respectively on the reported variables in Table 2.

**Table 2:** Organization of the variables and representation of the effect of the intervention

		22	27	28	31	37	23	33	32	24	29	34	26	30	35	36	25	Study **n**
		**ST**	**AE**	**AE**	**CO**	**FL**	**CO**	**CO**	**CO**	**ST**	**AE**	**CO**	**CO**	**AE**	**CO**	**CO**	**AE** **ST**	
**Productivity variables**	**Category 1: Workability**		+	+				+	+	=				+				5
	**Category 2: Absenteeism**				=	+		+	+				+		-		=	6
	**Category 3: Productivity**	+	=		=		=	+	+	=	+	+	+					9
**Health variables**	**Category 1: Health-related PA**					+	=	+	+	=					-		+	7
	**Category 2: Muscle strength**	+					+			+		+						4
	**Category 3: Body composition**	+		+	=			+	+			=	+			+		8
	**Category 4: Blood pressure**	+			=								+					3
	**Category 5: Musculoskeletal symptoms**	+	+				+										+	4
	**Category 6: Blood profile**				=			+	+									3
	**Category 7: Amount of PA**	+		+			=				+		+					5
	**Category 8: Cardiorespiratory capacity**			+			+	+	+			+		+	+			7
	**Category 9: Stress**				=											+	=	3
	**Category 10: Anxiety**					+						=				+		3

^a^*ST*, modality based on strength training

^b^*AE*, modality based on aerobic exercise

^c^*CO*, modality based on combination of aerobic and strength training

^d^*FL*, modality four based on flexibility or balance

^e^ + , represents when the intervention has a positive effect on the variable

^f^-, represents when the intervention has a negative effect on the variable; and

^g^ = represents when no changes in the variables are observed

## Results

**Fig. 1:**
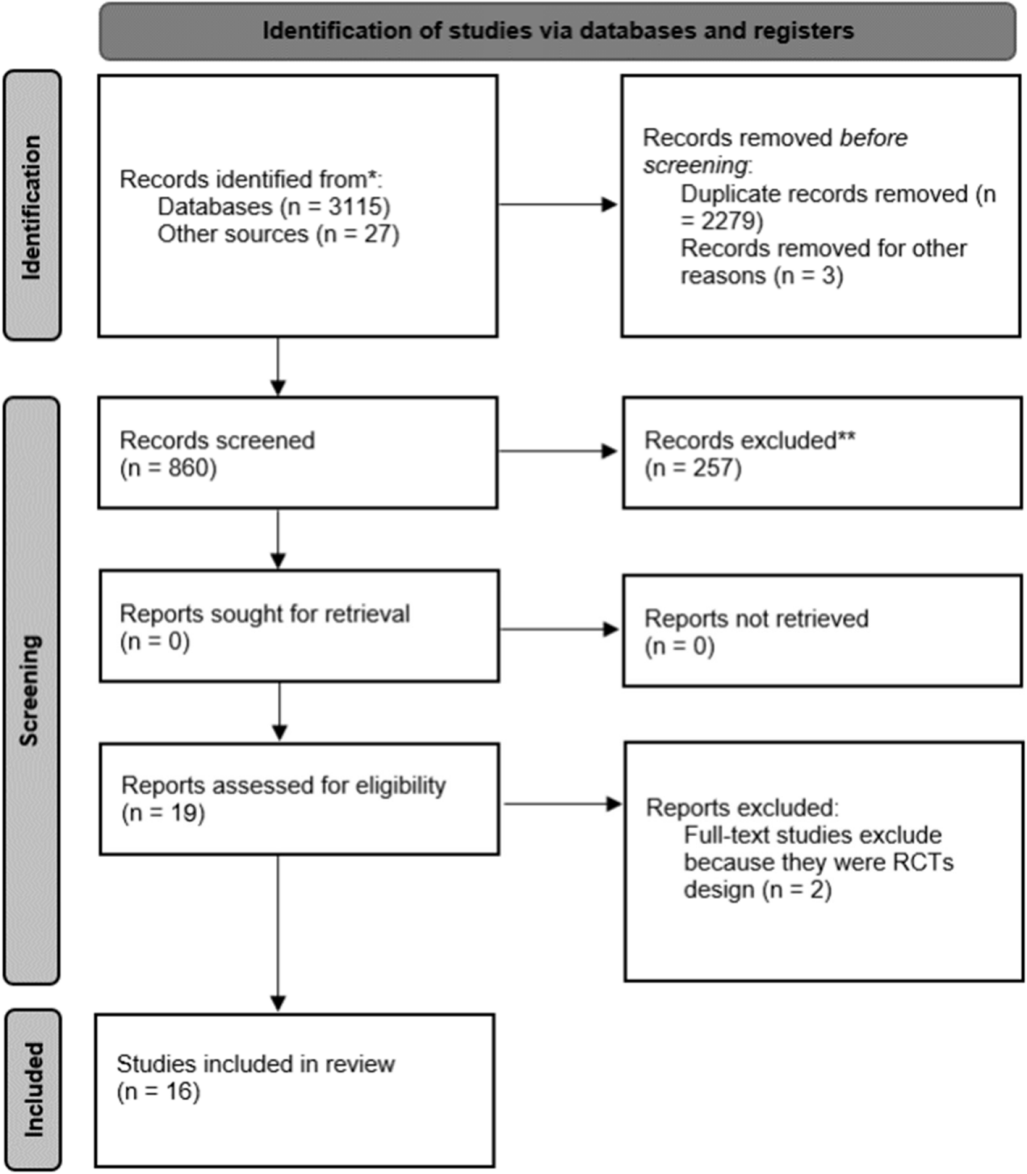
PRISMA 2020 flow diagram for new systematic reviews

Database searches yielded 3142 results, of which 2282 were duplicates. After removing duplicates, the database search identified 860 records, from which 603 full texts were assessed after title and abstract screening. Then, 585 studies were excluded due to not meeting the eligibility criteria, resulting in 19 studies. Two of these papers were eliminated because they were study protocol that did not report pre- and post-test results, and in one case, two separate publications originated from the same study. Therefore, this review included 17 publications from 16 studies. A PRISMA 2020 flow diagram of the study selection process is described in Fig. 1. A consensus discussion took place at the end of the data extraction process. Discrepancies and ambiguities were addressed by consulting two senior researchers (L. G. and J. F.) until full consensus was achieved. The most frequent reasons for exclusion were non-RCT designs and results not including both productivity and health outcomes. References of the population, intervention, exposure, and tests/outcomes for each article can be found on Supplementary Table S2: Description and characteristics of included studies. Finally, a variable was considered for the analysis when it reported *p*-value and allow the calculation of effect size, describing statics of pre and posttest in the intervention and control group. The specified statistics for each outcome used in the presentation of results is described on Supplementary Table S3: Statistical parameters of the variables analyzed.

### Description and characteristics of included studies

A total of 16 articles were published between 2002 and 2021. Five studies were performed in Denmark, two each in Norway and the UK, and finally, one each in Brazil, Germany, the USA, Spain, Japan, Netherlands, and Africa. Regarding the type of participants, five studies analyzed health care worker population, five studies focused their program on office workers, two studies focused on industrial workers, and two focused on university employees. Finally, one study each analyzed cleaners and post office workers.

### Interventions

In addition to the control group, four of the RCTs analyzed in this review included more than one intervention group which completed an exercise program [21–24]. This allowed the impact of different PA programs on various groups to be compared. Therefore, the interventions described below (*n* = 19) exceed the total number of studies included in the analysis (*n* = 16). As described above, the type of intervention was allocated into four different modalities based on the WHO classifications [11]: ST (*k* = 3) [21, 23, 24], AE (*k* = 5) [24–28], CO (*k* = 8) [22, 29–35], and FL (*k* = 1) [36].

Three studies included a nutrition component in addition to PA [24, 30, 34]. Ten of the studies delivered some form of education/counselling that included information about stress, coping, health, and nutrition, among others [21, 22, 24–27, 30, 34–36]. Four studies used a wearable pedometer or heart rate monitor to register the intensity of the intervention and to adapt the training to each participant [24, 26, 27, 34]. Only one study provided financial incentives for performing PA [30]. Four studies described environmental interventions [22, 27, 30, 34] such as a scan of environmental factors which may promote PA (e.g., high tables or gym on the workspace). Five studies described intervention which included techniques of behavior change based on productivity, stress coping, and teamwork [24, 30, 34–36]. In eight studies, the intervention was performed during both leisure time and working hours [21, 22, 26, 27, 30–32, 34]. Only two studies carried out the intervention only during leisure time [28, 36]. Meanwhile, six studies performed their PA intervention only during working hours [23–25, 29, 33, 35]. Finally, four articles included a follow-up analysis [24, 25, 28, 36].

### Variables analyzed in the studies

The variables described in Table 2 are based on quantifiable data measured in the studies.

#### Productivity variables

The variables reporting productivity effects were grouped into three categories as they were named in the articles.

- *Category 1*: Work-ability, an indicator based on the employee’s perception of their job performance and the estimation of their projection over the next 2 years [37], was measured through the workability index [23, 25, 26, 28, 31, 32].
- *Category 2*: Absenteeism, which refers to the temporary absence from work for reasons such as illness, death in the family, or other personal issues [38], was analyzed in six studies through the human resource department or self-reported questionnaire [24, 30, 32, 34, 36].
- *Category* 3: Productivity, an indicator that aims to measure worker efficiency [39], was analyzed in nine studies using the Health and Work Performance Questionnaire, the WHO Health and Work Performance Questionnaire, the Work Limitation Questionnaire, and self-reported questionnaires [21–23, 25, 27, 30–33].

#### Health variables

The variables reporting health effects were grouped into ten categories. *Category 1*: Health-related variables associated with health state self-perception. It was measured in 7 studies using self-reported health state questionnaire, the EQ-5D-5L, the health-related quality of life, the COOP/WONCA charts, and subjective ad hoc questionnaire [22–24, 31, 32, 34, 36]. *Category 2*: Muscle strength was analyzed in four articles through different tests such a one maximum repetition (1 RM), test of maximal voluntary isometric muscle strength, 90° push-up test, and standing long jump test [21–23, 33]. *Category 3*: Body composition (BMI, body weight (kg), muscle mass percentage (%), and body fat percentage (%)) were measured in 8 studies using bioimpedance, scales, and stadiometers. *Category 4*: Blood pressure [21, 29, 30] and *category 5*: blood profile variables (total cholesterol, fasting blood glucose, triglycerides) were measured in three studies each [30–32]. *Category 6*: Musculoskeletal symptoms (pain, perceived risk, rating of perceived exertion) were measured in four studies through self-reported questionnaires or specific protocols such a Roland-Morris Disability Questionnaire, the Borg’s scale for physical exertion, Nordic Musculoskeletal Questionnaire, a 5-step ordinal scale and need for recovery scale, and the Health Complaints Inventory [21, 22, 24, 25, 28]. *Category 7*: Amount of PA (PA total score (MET-h/week) and regular exercise (days/week of moderate exercise, number of hours sitting per day, and daily step count) were analyzed in 5 studies using questionnaires and tools like the Baecke PA Questionnaire, Freiburger PA Questionnaire, IPAQ Questionnaire, self-reported questionnaires, accelerometer, and pedometers [21, 22, 26, 27, 29, 30]. Finally, *category 8*: cardiorespiratory capacity variables were measured 7 times using a submaximal incremental bicycle exercise test, the Åstrand 1-point sub-maximal test on a bicycle, 20-m shuttle run test, Urho Kaleva Kekkonen walk test (VO2max), and the UKK fitness test score (aerobic fitness) [22, 23, 26, 28, 31–34]. *Category 9*: Stress, defined as the physical and mental responses of the body and the adaptations to perceived changes in life [40], was measured in four studies using the Job Stress Questionnaire, the Cooper Job Stress questionnaire, and self-report questionnaires [24, 29, 30, 35]. Finally, *category 10*: anxiety, a psychological and physiological state characterized by feelings of apprehension, motor tension, and autonomic overactivity that blocks and limits work abilities [41], was analyzed three times with the Keele STarT Back Screening Tool and self-report anxiety questionnaires [33, 35, 36].

#### Economic variables

Only one study included economic measured the quality of life (HRQL) assessed with the EQ-5D-5L and quality-adjusted life-years (QALYs) [36].

### Effect size

For each study, we calculated effect sizes to enhance the comparability of included studies using standardized mean differences (Cohen’s d) for each outcome variable. For three studies, the standardized effect size could not be calculated due to insufficient data. Therefore, a total of 58 post hoc standardized mean differences are included (see Supplementary Table S3: Statistical parameters of the variables analyzed). Twenty-eight outcomes had small effect sizes (*d* < 0.20), twenty-three were medium effect sizes (*d* = 0.20–0.50), and seven were large effect sizes (*d* =  > 0.80). The median effect size was 0.40 (interquartile range 0.07–0.4). A meta-analysis with sample size weighting was not feasible due to the scope of this review, which includes differing health conditions, participant work status, study designs, and outcome measures, as well as the level of detail reported. Therefore, a best-evidence synthesis approach was considered better suited for this study.

### Risk of bias

**Fig. 2:**
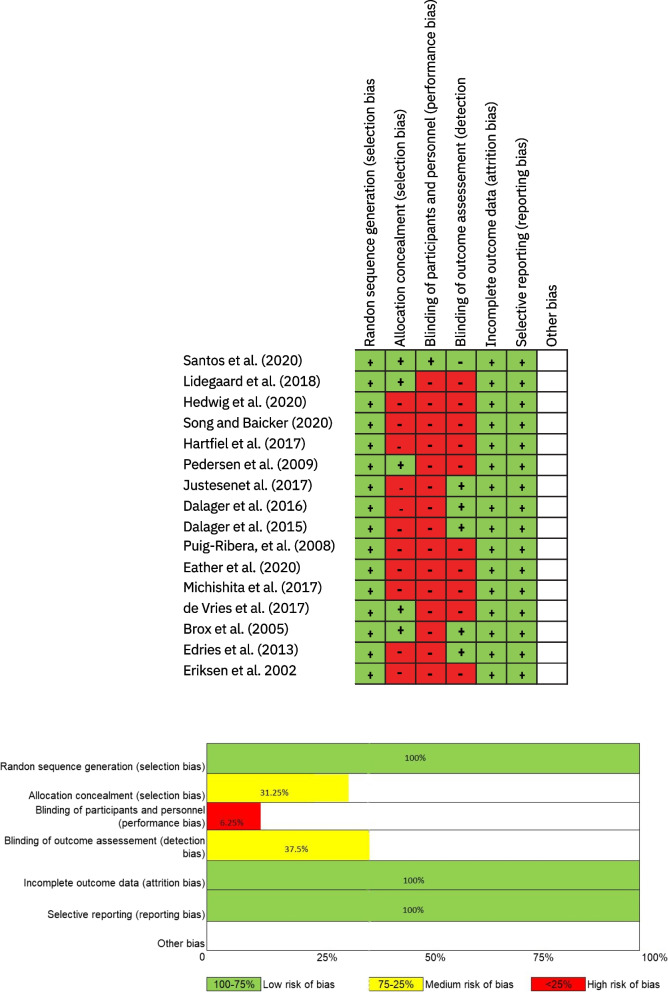
Estimated risk of bias across all studies. Risk-of-bias summary and graph with detailed assessments using the Cochrane Collaboration tool. Each domain was judged as high, low, or unclear risk of bias with the overall assessment of each study graded as low risk of bias (when more than five domains were low risk of bias), high risk of bias (at least three domains were high risk of bias), or medium risk of bias (otherwise). Because most of the included studies were well-designed RCTs, most of them were assessed as low risk of bias

All studies were RCTs as defined in the eligibility criteria. Other study designs such a pre-post-design, cohort study, or quasi-experimental study were excluded. The Cochrane Handbook classification guide was followed, with reviewers assigning high-, medium-, or low-risk level to studies in terms of seven types of bias: (i) random sequence generation (selection bias), (ii) allocation concealment (selection bias), (iii) performance (blinding of participants and personnel), (iv) detection (blinding of outcome assessment), (v) attrition (incomplete outcome data), (vi) reporting (selective reporting), and (vii) other bias. According to the classification, 100% of the studies randomized their participants, while 31.25% concealed the allocation. Only 6.25% of the studies blinded the participant and the examiner. The inability to blind the participants introduces multiple risks of nonspecific effects, including possible placebo effects in respect of changes in the selected outcomes, as well as the possibility of a Hawthorne effect [42]. However, due to the type of intervention or the content of the PA training intervention, the participants and the instructors supervising the program could not be blinded (more information in the interventions section). Finally, 37.50% blinded the outcome assessment. The results of the risk-of-bias analysis for all studies are displayed in Fig. 2.

## Discussion

This is the first systematic review to (1) analyze the effectiveness of WPPAs by PA modality (AE, ST, FL, or CO) on both productivity and health outcomes of workers in RCTs and (2) assess the economic impact of these programs. The main findings were that all types of WPPAs analyzed (AE, ST, FL, or CO) improved both workers’ productivity and health. It was not possible to make strong conclusion about each modality’s effectiveness because the studies displayed a large heterogeneity in duration, working population, and methodology; also, only one study compared two types of WPPAs (AE vs ST) [24]. Finally, the economic impact could not be analyzed either because only one study reported this data [36].

### Impact of WPPAs on productivity

Most of the variables showed positive changes after the application of WWPA programs, while one study reported a deterioration on two productivity variables after the intervention. However, the use of different tools to measure productivity variables limited our ability to determine which WWPA is better for improving productivity.

Workability increased in 5 out of 6 studies (2/2 CO, 3/3 AE, and 0/1 ST), showing that WPPAs programs are effective in improving this variable in different working populations, as previously suggested by a meta-analysis [43]. Only one study did not show changes in workability after a 12-month WWPA based on ST [23]. However, the baseline values in this study were very high (9.2 out of 11), which could create a ceiling effect and explain the lack of improvements after this intervention. Interventions based on AE of 20–60 min for at least 2–3 times per week, and at 60% of the VO2max, are effective in improving workers workability [25, 26, 44]. On the contrary, the heterogeneity of CO programs does not allow for guidance on intensity, duration, type of exercise, and frequency, although 12 months of intervention seem effective to see changes in workability [31, 32].

Occupational environment that does not promote PA has been identified as a potential risk factor for absenteeism [45, 46]. This systematic review confirms that WWPA can reduce absenteeism, although only 3 out of 6 studies reported improvements (2/4 CO, 1/1 FL, and 0/1 AE-ST). The 3 studies that did not report improvement in absenteeism [24, 34, 47] evidence that measuring this variable is not easy because there are different factors that influence absenteeism, such as family member sickness, civic duties, type of job, income, and workplace environment. Furthermore, social class and workplace benefits can also influence the absence rate and limit the efficacy of WPPAs programs that are implemented in different work settings [47].

Finally, in line with previous research [48], which suggest that workers can improve their work performance by taking part in WPPAs, productivity increased after WPPAs in several studies (3/5 CO, 1/2 ST, and 1/2 AE). Although the four studies that did not report changes in productivity might suggest a limited ability of WPPAs programs, they all displayed a high baseline values [22, 23, 25, 47], and the room for improvement is limited. On the other hand, the CO programs including 1–3 sessions per week, a duration between 10 to 60 min with high-intensity exercises [31–33], AE based on walking program [27], and ST based on resistance training [21] seem to be effective in improving worker productivity, but further research is needed.

### Impact of WPPAs on health

One of the main findings of this review is that WPPAs programs improve many different health-related variables in workers from different work settings, jobs characteristics, etc. This is in line with the existing evidence, which suggests that semi- and structured PA are beneficial for improving workers health [49].

This review shows that WPPAs are effective in improving cardiorespiratory capacity (2/2 AE and 5/5 CO), muscle strength (2/2 ST and 2/2 CO), and musculoskeletal symptoms (2/2 AE, 1/1 ST, 1/1 CO, and 1/1 AE-ST), regardless of different types of PA program, were applied. The improvements in cardiorespiratory fitness through WPPAs are in line with other authors [50], who suggested that moderate and vigorous PA from WPPAs (running, cycling, walking, rowing, and dancing) can improve cardiorespiratory fitness [22, 26, 28, 31–34]. Furthermore, the greater increment in cardiorespiratory fitness reported in higher activity level groups compared to the low activity level groups or reference group is consistent with the existing literature [51]. Increments in muscle strength have been observed with strength exercises including dumbbells, elastic band and barbells, isometric exercises, and HIIT. Furthermore, in line with the existing literature [52, 53], this type of exercise also revealed improvements in productivity and musculoskeletal diseases [21–23, 33]. Finally, the statement of PA is a tool to prevent musculoskeletal disability at the workplace [54] which is confirmed by the positive improvements reported in selected studies. Accordingly, Keele STarT Back Screening Tool CO, AE, and ST can be useful to address variables associated with musculoskeletal symptoms such as fatigue, exertion, postural control, or musculoskeletal pain symptom [21, 22, 24, 25].

The remaining health-related variables did not improve after all the analyzed interventions, but they did not get worsen either. Blood pressure (1/1 ST and 1/2 CO) [21, 22] and cholesterol (2/3 CO) [31, 32] improved in 2 out of 3 studies analyzed. This fact suggests that WPPAs programs can be beneficial in addressing cardiovascular risk factors as reported in a previous meta-analysis [14]. However, not all proposed PA stimuli are sufficient to cause improvement in this variable. The intensity, volume, and days/week should be considered. In fact, an unsuitable design might explain the lack of changes in the clinical measures of health included in this analysis [30].

The amount of PA measured in days/week, moderate or vigorous activity, training volume, number of steps per day, and PA level measured through questionnaires, accelerometers, or pedometers improved on 4 out of 5 studies (1/2 CO, 2/2 AE, and 1/1 ST) [21, 22, 26, 27, 29, 30]. Only one study did not describe any change after the CO program. Nonetheless, the inherent limitations associated with the IPAQ questionnaire in detecting relatively small changes in PA, as the WPPAs included 1 h/week of PA at work [22]. Additionally, a high percentage of participants in this study self-reported high activity levels at baseline, which may partly explain the lack of significant change in the level of PA.

Body composition improved in 6 out of 8 studies (4/6 CO, 1/1 ST, and 1/1 AE) [21, 23, 24, 26, 29, 32, 55], showing the effectiveness of WPPAs in improving this variable. Two studies did not report any changes after the intervention. However, the duration of the WPPAs (8 weeks) [33], the lack of supervision, and the absence of counseling on nutritional issues [30] might explain the limited changes in healthy adult populations [56].

Self-perceived ratings of health showed improvement in 4 out of 7 studies (2/4 CO, 1/1 FL, 0/1 ST, and 1/1 AE-ST) [24, 26, 32, 36, 55], showing that WPPAs are able to improve employees’ overall lifestyle habits, decrease perceived fatigue, and increase willingness and readiness for their jobs [31]. Two interventions did not report any changes (1 ST and 1 CO) [22, 23]. However, they reported high values of workers’ self-reported health at baseline, what might explain the absence of significant improvements after the intervention [22, 23].

In a previous systematic review, lower values of work-related stress were associated with WPPAs [57]. This paper is partially in line with this previous study as 1 of 3 studies (1/2 CO and 0/1 AE-ST) showed improved workers’ stress levels [29, 35]. However, none of the selected studies used objective tools for measuring stress (e.g., heart rate variability, saliva), only self-reported questionnaires [58]. Based on the selected papers, CO-based programs of 10–30 min per day, aerobic exercise, or core training at low to moderate intensity seems to be enough to produce improvements in stress, although further research is needed. One study found no improvement on self-reported stress using the Cooper job stress questionnaire after a AE or a ST program, but most participants stated that the intervention was effective in improving their mental health and stress in responses to qualitative questions [24].

Finally, this review provides evidence that workers’ anxiety can be reduced through WPPAs, with 2 of 3 studies finding improvements (1/2 CO and 1/1 FL). Nonetheless, the lack of effectiveness reported is likely to be due to the limitations of methods they used to measure anxiety [33]. Anxiety is a complex variable that might be conditioned by the interaction of the individual with different environmental factors, and this interaction was not assessed in this study. Further research is needed to set guidelines on PA characteristics.

### Effectiveness of the type of intervention

Given the heterogeneity of the WPPAs analyzed (duration, frequency, working population, type of intervention), it is not possible to determine what type of intervention (AE, ST, CO, or FL) is the most effective to improve both productivity and health of workers. Furthermore, although none of the studies analyzed included an intervention based on a comprehensive model (e.g., TWH), many of them included multiple components (education, rewards, counseling, nutrition, etc.). This makes it difficult to associate the effects of the intervention solely to the PA program. This limitation should be addressed in future studies as it would permit to design of more effective WPPAs.

### Cost-effectiveness analysis

Only one study included a cost-effectiveness analysis. Results of this study showed improvement in health-related quality of life, reduced lower back pain and absenteeism, and concluded that the probability of the program being cost-effective was 95% [36]. Future studies should include this variable in order to understand the ROI that this kind of program has on the company that implement them. It would also help to design more cost-effective WPPAs.

## Conclusions

WPPAs are effective to improve both productivity and health of workers. Health-related variables such as cardiorespiratory fitness, muscle strength, and muscular ability increased in all the studies in which they were included. Concerning productivity variables, workability is the most positively affected after WPPAs. The heterogeneity of the studies (duration, type of working population, intensity, frequency, type of exercise, etc.) does not permit to study of which modality (AE, ST, CO, or FL) is more effective. Finally, most WPPAs programs reported in the literature do not analyze the economic return of these programs. Furthermore, this review provides a comprehensive framework on different tools to measure the target outcomes related to WPPAs. However, the need for a gold standard of measurement tools has been highlighted, and the necessity of integrating economic analyses in this type of intervention is also noteworthy, as they could provide more concrete benefits to work environments. Finally, this review emphasizes the need to deepen the examination of WPPAs by different modalities, and specific, targeted programs for different working populations could be used. As a recommendation, practitioners and researchers should carefully examine the company objectives and target sample characteristics to implement the most appropriate WPPA in terms of duration, intensity, and modality (AE, ST, CO, or FL). In addition, this systematic review is a helpful resource for finding out the health and productivity indicators used in WPPAs.

### Limitations

A limitation of the current review is that we were not able to pool the data for a formal meta-analysis with sample size weighting. There is no gold standard strategy for measuring the productivity or health variables in ways that are meaningful to employees and to organizations. Selected studies used a large variety of tools and protocols, mixing both objective and subjective evaluations. Also, conclusions are limited as included studies targeted different worker populations, were implemented in different working settings, and applied a different WPPAs, so factors such as physical requirements of each job, incomes, or working conciliation may influence the variables analyzed. Finally, the lack cost-effectiveness analysis in the selected programs did not permit economic analyses.

## Supplementary Information

**Additional file 1: Supplementary Table S1.** Databases search strategy. DATABASE: PUBMED. DATABASE: WEB OF SCIENCE. DATABASE: SCOPUS. DATABASE: MEDLINE. DATABASE: SPORTDISCUS.

**Additional file 2: Supplementary Table S2.** Description and characteristics of included studies(27,28,30)(26,27,29).

**Additional file 3: Supplementary Table S3.** Statistical parameters of the variables analyzed.

## Data Availability

The datasets supporting the conclusions of this article are included within the article and its additional files Supplementary Table S1: Databases search strategy; Supplementary Table S2: Description and characteristics of included studies; Supplementary Table S3: Statistical parameters of the variables analyzed, and the *PRISMA 2020 checklist.*

## Abbreviations

AE: Intervention based on aerobic exercise
ST: Intervention based on strength exercise
FL: Intervention based on flexibility or balance exercise
CO: Intervention that combined aerobic and strength exercise
WWPs: Worksite wellness programs
PA: Physical activity
TWH: Total Worker Health®
WPPAs: Worksite program based on physical activity
