# Supplementary material for: Effectiveness of worksite wellness programs based on physical activity to improve workers’ health and productivity: a systematic review
Source: Syst Rev. 2023 May 24;12:87. doi: 10.1186/s13643-023-02258-6 (PMC10207792; doi:10.1186/s13643-023-02258-6)
Supplement: Supplementary file 1 — Additional file 1: Supplementary Table S1. Databases search strategy. DATABASE: PUBMED. DATABASE: WEB OF SCIENCE. DATABASE: SCOPUS. DATABASE: MEDLINE. DATABASE: SPORTDISCUS. [file 13643_2023_2258_MOESM1_ESM.docx]

**Supplementary table S1**: databases search strategy

DATABASE: PUBMED

|  | AND | | | | | n | Cost-effectiveness | n | Cost-benefit | n |
| --- | --- | --- | --- | --- | --- | --- | --- | --- | --- | --- |
| Combination 1 | workplace | employee | physical activity | Productivity | health | 55 | Combination 1.1 | 16 | Combination 1.2  N= 13 | 13 |
| Combination 2 | worksite health promotion program | employee | physical activity | productivity | health | 30 | Combination 2.1 | 10 | Combination 2.2  N=9 | 9 |
| Combination 3 | workplace | worker | physical activity | Productivity | health | 46 | Combination 3.1 | 9 | Combination 3.2  N= 7 | 7 |
| Combination 4 | worksite health promotion program | worker | physical activity | productivity | health | 18 | Combination 4.1 | 6 | Combination 4.2.  N=5 | 5 |
| Combination 5 | workplace | employee | exercise | Productivity | health | 42 | Combination 5.1 | 9 | Combination 5.2  N= 7 | 7 |
| Combination 6 | worksite health promotion program | employee | exercise | productivity | health | 24 | Combination 7.1 | 7 | Combination 7.2  N=7 | 7 |
| Combination 7 | workplace | worker | exercise | Productivity | health | 36 | Combination 3.1 | 5 | Combination 3.2  N= 4 | 4 |
| Combination 8 | worksite health promotion program | worker | exercise | productivity | health | 24 | Combination 4.1 | 8 | Combination 4.2. | 7 |
| Total studies: 404 | | | | | | 275 |  | 70 |  | 59 |

DATABASE: WEB OF SCIENCE

|  | AND | | | | | n | Cost-effectiveness | n | Cost-benefit | n |
| --- | --- | --- | --- | --- | --- | --- | --- | --- | --- | --- |
| Combination 1 | workplace | employee | physical activity | Productivity | health | 157 | Combination 1.1 | 9 | Combination 1.2 | 9 |
| Combination 2 | worksite health promotion program | employee | physical activity | productivity | health | 38 | Combination 2.1 | 4 | Combination 2.2 | 1 |
| Combination 3 | workplace | worker | physical activity | Productivity | health | 169 | Combination 3.1 | 8 | Combination 3.2  N= 7 | 9 |
| Combination 4 | worksite health promotion program | worker | physical activity | productivity | health | 22 | Combination 4.1 | 2 | Combination 4.2.  N=5 | 2 |
| Combination 5 | workplace | employee | exercise | Productivity | health | 106 | Combination 5.1 | 4 | Combination 5.2  N= 7 | 2 |
| Combination 6 | worksite health promotion program | employee | exercise | productivity | health | 13 | Combination 6.1 | 3 | Combination 6.2  N=7 | 0 |
| Combination 7 | workplace | worker | exercise | Productivity | health | 45 | Combination 7.1 | 2 | Combination 7.2  N= 4 | 2 |
| Combination 8 | worksite health promotion program | worker | exercise | productivity | health | 8 | Combination 4.1 | 1 | Combination 4.2. | 0 |
| Total studies: 616 | | | | | | 558 |  | 33 |  | 25 |

DATABASE: BASE DE DATOS SCOPUS

|  | AND | | | | | n | Cost-effectiveness | n | Cost-benefit | n |
| --- | --- | --- | --- | --- | --- | --- | --- | --- | --- | --- |
| Combination 1 | workplace | employee | physical activity | Productivity | health | 108 | Combination 1.1 | 8 | Combination 1.2 | 6 |
| Combination 2 | worksite health promotion program | employee | physical activity | productivity | health | 18 | Combination 2.1 | 3 | Combination 2.2 | 1 |
| Combination 3 | workplace | worker | physical activity | Productivity | health | 108 | Combination 3.1 | 3 | Combination 3.2  N= 7 | 4 |
| Combination 4 | worksite health promotion program | worker | physical activity | productivity | health | 9 | Combination 4.1 | 0 | Combination 4.2.  N=5 | 1 |
| Combination 5 | workplace | employee | exercise | Productivity | health | 80 | Combination 5.1 | 3 | Combination 5.2  N= 7 | 2 |
| Combination 6 | worksite health promotion program | employee | exercise | productivity | health | 14 | Combination 6.1 | 1 | Combination 6.2  N=7 | 0 |
| Combination 7 | workplace | worker | exercise | Productivity | health | 73 | Combination 7.1 | 1 | Combination 7.2  N= 4 | 3 |
| Combination 8 | worksite health promotion program | worker | exercise | productivity | health | 5 | Combination 8.1 | 0 | Combination 8.2. | 0 |
| Total studies: 451 | | | | | | 415 |  | 19 |  | 17 |

DATABASE: MEDLINE

|  | AND | | | | | n | Cost-effectiveness | n | Cost-benefit | n |
| --- | --- | --- | --- | --- | --- | --- | --- | --- | --- | --- |
| Combination 1 | workplace | employee | physical activity | Productivity | health | 209 | Combination 1.1 | 87 | Combination 1.2 | 61 |
| Combination 2 | worksite health promotion program | employee | physical activity | productivity | health | 23 | Combination 2.1 | 8 | Combination 2.2 | 8 |
| Combination 3 | workplace | worker | physical activity | Productivity | health | 216 | Combination 3.1 | 84 | Combination 3.2  N= 7 | 50 |
| Combination 4 | worksite health promotion program | worker | physical activity | productivity | health | 22 | Combination 4.1 | 8 | Combination 4.2.  N=5 | 8 |
| Combination 5 | workplace | employee | exercise | Productivity | health | 213 | Combination 5.1 | 90 | Combination 5.2  N= 7 | 65 |
| Combination 6 | worksite health promotion program | employee | exercise | productivity | health | 16 | Combination 6.1 | 8 | Combination 6.2  N=7 | 8 |
| Combination 7 | workplace | worker | exercise | Productivity | health | 217 | Combination 7.1 | 84 | Combination 7.2  N= 4 | 62 |
| Combination 8 | worksite health promotion program | worker | exercise | productivity | health | 18 | Combination 8.1 | 8 | Combination 8.2. | 8 |
| Total studies: 1581 | | | | | | 934 |  | 377 |  | 270 |

DATABASE: SPORTDISCUS

|  | AND | | | | | n | Cost-effectiveness | n | Cost-benefit | n |
| --- | --- | --- | --- | --- | --- | --- | --- | --- | --- | --- |
| Combination 1 | workplace | employee | physical activity | Productivity | health | 23 | Combination 1.1 | 1 | Combination 1.2 | 0 |
| Combination 2 | worksite health promotion program | employee | physical activity | productivity | health | 2 | Combination 2.1 | 0 | Combination 2.2 | 0 |
| Combination 3 | workplace | worker | physical activity | Productivity | health | 13 | Combination 3.1 | 1 | Combination 3.2  N= 7 | 0 |
| Combination 4 | worksite health promotion program | worker | physical activity | productivity | health | 6 | Combination 4.1 | 0 | Combination 4.2.  N=5 | 0 |
| Combination 5 | workplace | employee | exercise | Productivity | health | 17 | Combination 5.1 | 2 | Combination 5.2  N= 7 | 2 |
| Combination 6 | worksite health promotion program | employee | exercise | productivity | health | 4 | Combination 6.1 | 0 | Combination 6.2  N=7 | 0 |
| Combination 7 | workplace | worker | exercise | Productivity | health | 14 | Combination 7.1 | 2 | Combination 7.2  N= 4 | 1 |
| Combination 8 | worksite health promotion program | worker | exercise | productivity | health | 2 | Combination 8.1 | 0 | Combination 8.2. | 0 |
| Total studies: 90 | | | | | | 81 |  | 6 |  | 3 |
