# Supplementary material for: Effectiveness of worksite wellness programs based on physical activity to improve workers’ health and productivity: a systematic review
Source: Syst Rev. 2023 May 24;12:87. doi: 10.1186/s13643-023-02258-6 (PMC10207792; doi:10.1186/s13643-023-02258-6)
Supplement: Supplementary file 2 — Additional file 2: Supplementary Table S2. Description and characteristics of included studies(27,28,30)(26,27,29). [file 13643_2023_2258_MOESM2_ESM.docx]

| **Table S2.** Description and characteristics of included studies | | | | | | | | | | | | | | | |  |
| --- | --- | --- | --- | --- | --- | --- | --- | --- | --- | --- | --- | --- | --- | --- | --- | --- |
| **Reference** | **Sample (n);**  **Age (years);**  **Sex (M/F)** | **Intervention Duration** | **Intervention Type** | **Setting type of industry** | **Aim of the study** | **Type of intervention** | **PA Protocol description** | **Productivity Outcomes** | **Health Outcomes** | **Economical outcomes** | | **Conclusion** | |  | |  |
| (Santos et al., 2020)^21^ | 204;  Intervention group (98)  34.3  F(20);M(78); Control group (106)  37.8  F(14);M(92) | 4 months | RCT | Industrial workers | To evaluate the effects of a resistance exercise program on perceived fatigue control among industrial workers | *Intervention group:*  Progressive resistance exercise (PRE)  *Control group:*  Compensatory workplace exercise (CWE) | (PRE): 30% of the one-repetition maximum (1 RM - kg). using dumbbells and barbells  (CWE): during breaks stretching and elastic bands | - Productivity (The Health and Work Performance Questionnaire) | - Musculoskeletal symptoms (Need for Recovery Scale and Nordic Musculoskeletal Questionnaire) - Muscle Strength (A maximum repetition, 1RM) - Amount of PA (The Baecke PA Questionnaire) - BMI (Body mass Index) - Blood pressure |  | | Both programs were effective PRE and CWE. However, CWE during work breaks presented better results on all measured outcomes | |  | |  |
| (Lidegaard et al., 2018)^22^ | 116;  Intervention group (57)  44.9  F(43);M(14); Control group (59)  45.7  F(45);M(14) | 12 months | RCT | cleaners | This study assessed the effects of a worksite aerobic exercise intervention on: work-ability, need for recovery, productivity, and rating of exertion | *Intervention group:*  aerobic exercise was offered 2 weekly aerobic exercise sessions of 30 min over the entire 12-month intervention period  *Control group:* receiving promoting lectures | The aerobic exercise sessions targeted an average intensity of minimum 60% of maximal oxygen consumption | - Work-ability (Work Ability Index questionnaire, WAI) - Productivity (the WHO Health and Work Performance Questionnaire) | - Musculoskeletal symptoms (Rating of perceived exertion, the Borg’s scale for physical exertion) |  | | After 12 months’ work-ability improved and need for recovery decreased | |  | |  |
| (Stenner et al., 2020)^23^ | 265  F  Intervention group (146)  Control Group (145) | 6  months | RCT | hospital workers of various occupations. | To test the effects of guided endurance training on work-ability in middle-aged female hospital workers of various occupations | *Intervention group*: 210 min of endurance training a week (20–60 min units for at least 3 days per week.  *Control group:*  No intervention. | The participants received heart rate ranges based on the lactate threshold (approx. 60–80% of the estimated max)  activities: cycling, rowing and walking. | - Work-ability (WAI) | - Cardiorespiratory capacity (incremental bicycle exercise test, VO2max) - BMI, fat- and fat-free mass. - Amount of PA (Freiburger PA Questionnaire, MET-h/wk). |  | | A 6-month exercise intervention with individualized endurance activities and group fitness courses in middle aged female hospital workers resulted in positive effects on work-ability and cardiorespiratory fitness. The support of employees with the weakest work-ability could be a beneficial strategy for long-term work-ability. | |  | |  |
| (Song & Baicker, 2020)^24^ | 32.974;  Intervention group (4037)  F(46%);M(54%);  Control group (28937)  F(54%);M(46%) | 18 months | RCT | varied | To evaluate a multicomponent workplace wellness program resembling programs offered by US employers. | *Intervention group:*  The program comprised 8 modules focused on nutrition, physical activity, stress reduction, and related topics.  *Control group:*  Control worksites received no wellness programming. | Modules 3 and 4: Club Cardio Challenge Rounds.  20 minutes or more of cardiovascular exercise at least 3 days per week  Module 7: Weight Loss Boot Camp. Exercising for at least 30 minutes  Module 8: Movin’ in May. Exercise for at least 30 minutes 3 days per week and track their exercise | - Absenteeism: (self-reported days off) - Productivity: self-reported Performance questionnaire | - Blood profile (cholesterol, HDL and glucose) - BMI - Blood preassure (Systolic and Diastolic preassure) - Amount of PA (days/week of moderate exercise, %; nº of hours sitting per day) - Stress: self-repored stress questionnaire |  | | There were no significant differences in any variable. | |  | |  |
| (Hartfiel et al., 2017)^25^ | 151;  Intervention group (76)  F (70); M (6)  Control group (75)  F (70); M (5) | 2 months | RCT | hospital workers of various occupations. | To assess the cost-effectiveness of yoga for managing musculoskeletal conditions | *Intervention group:*  The participants received an specific intervention based on yoga characterized by specific movements, directed breathing, and relaxation methods that include affirmation and visualization techniques. Yoga participants also received a DVD and an illustrated booklet for home practice  *Control group:*  Usual-care participants received two evidence-based booklets: The Back Book and How to Manage Stress. | One free 60-minute session per week for 8 weeks. Four stages: activation exercises, energy block release sequences, back care postures and relaxation techniques | - Absenteeism (total days) | - Musculoskeletal symptoms (Roland-Morris Disability Questionnaire) - Anxiety (Keele STarT Back Screening Tool) | | QALYs (sickness absence day). | | Yoga is associated with improved health-related quality of life, and thus abseentism decrease. The intervention is likely to be cost-effective. | |  | |
| (Pedersen et al., 2009)^26^ | 549;  SRT group (180)  APE group (187)  REF group  (182) | 12 months | RCT | Office workers | To investigate the effect of two contrasting physical activity worksite interventions versus a reference intervention (REF) on various health outcomes. | *Intervention groups:*   - SRT consisted of traditional dynamic strengthening exercises. - PE consisted of various types of physical activities at the worksite.   *Control group:*   - REF group were encouraged to improving their knowledge on health. | SRT were performed with dumbbells for the muscles of the shoulder girdle and isometric exercises for the muscles of the cervical spine.  PE: Nordic Walking group | - Productivity (an 11- step ordinal scale: “How do you perceive your overall productivity the last 4 weeks?) | - Amount of PA (The International Physical Activity Questionnaire, IPAQ, MET h/wk) - Muscle Strength (Test of maximal voluntary isometric muscle strength) - Cardiorespiratory capacity (VO2max, a submaximal cycle ergometer test) - BMI - Blood Pressure - Musculoskeletal symptoms (a 5-step ordinal scale) |  | | The interventions resulted in clinically relevant effects on musculoskeletal pain as well as systolic blood pressure at 1 year, and body fat percentage at 6 months.  No significant changes in self-related productivity and general health were noted probably due to high levels at baseline. | |  | |  |
| (Dalager et al., 2016; Justesen et al., 2017)^27,28^ | 379;  44+-10;  F (74%)  M (26%)  Intervention group (N= 193) Control group (N= 194) | 24 months | RCT | Office Workers | To investigate the effect of individually tailored intelligent physical exercise training (IPET) | *Intervention group:*  Training group received high-intensity IPET within working hours. TG was recommended to perform moderate-intensity physical activity during leisure time.  *Control group:*  no wellness programming. | - 1-hour high-intensity IPET once a week. - 30 minutes of moderate-intensity physical activity (PA) 6 days a week during leisure-time. | - Work-ability (WAI) - Productivity (was rated on a 10-step ordinal scale: How do you perceive your overall productivity for the last 3 months?) - Absenteeism (number of days, from Human Resources managers) | - Cardiorespiratory capacity (VO2max, Åstrand one-point sub-maximal test on a bicycle) - BMI, Body mass, fat percentages and muscle mass - Blood profile (fasting blood glucose, triglycerides, low density lipoprotein (LDL) and high density lipoprotein (HDL)). - Health related PA (a self-reported general health was rated on a five-step nominal scale: How do you rate your health, all in all?) |  | | Implementing IPET during working hours for 1 year among office workers was the significant increase in productivity and general work-ability.  The intervention significantly improved work-ability, general health, CRF and systolic blood pressure  for the TG.  This implies that IPET has the potential to decrease SA. | |  | |  |
| (Dalager et al., 2015)^29^ | - 1 WS Intervention group= 116 - 3WS Intervention group= 126 - 9WS Intervention group= 106 - 3MS Intervention group= 124 - Control group= 101   573;  F (351)  M (222) | 12 months | RCT | Office workers | To determine the effect of strength training within workinghours, performed with the same total training volume but with different training frequencies. | *Intervention groups:*  The four training groups (1WS; 3WS;9WS;3MS) were scheduled to perform the same total amount of exercises (nine) and repetitions per week. The training groups differed on the frequency and the duration of each single training session.  *Control group:*  REF: a reference group without training. | - 1WS had one 60-min training session per week with supervision - 3WS: three 20-min supervised sessions/week - 9WS: nine 7-min supervised sessions/week - 3MS: three20-min sessions/week with minimal supervision - REF: a reference group without training. | - Work-ability (WAI) - Productivity (11-step numerical rating scale: How do you perceive your overall productivity the last four weeks?) | - Health related PA (a 5-step ordinal scale : How do you perceive your overall health?) - Muscle strength (1 RM) |  | | The training groups improved significantly their muscle strength and endurance both being significant compared with REF. | |  | |  |
| (Puig-Ribera et al., 2008)^30^ | 70;  Intervention group Walking Routes (WR)= n= 26; Intervention group Walking in task (WT) = 26 Control group (N= 27)  39±8;  F=57  M=22 | 2 months and 1 week | RCT | University employees | To compare the impact of walking programmes across university employees | *Intervention group:*   - Walking routes: - Walking while working:   *Control Group:*  No intervention | - Walking routes: To provide a map with some examples of walks around campus, with participants asked to complete at least 15 minutes of continuous, brisk walking every workday. - Walking while working: group´s guide encouraged accumulation of step counts targeting the office, lectures and seminars as contexts where task could be completed standing and walking, rather than sitting. | - Productivity (Work limitation Questionnaire) | - Amount of PA (Step counts with pedometers) |  | | Initially low active participants showed the greatest increase in step counts and improved QoL and work productivity.  These data indicate the potential for improving QoL and job productivity through workplace walking in inactive employees. | |  | |  |
| (Eather et al., 2020)^31^ | 47  43.0 ± 10.7  F=41  M=6  Intervention group (N= 24) Control group (N= 23) | 5 months | RCT | University employees | To assess the efficacy of a high-intensity interval training (HIIT) intervention in the workplace on physical and mental health outcomes. | *Intervention group:*  HIIT sessions  *Control group:*  No intervention | Sessions included a 2-minute gross-motor warm-up, followed by various combinations of aerobic and muscular fitness exercises lasting 8 minutes (using 30:30 second work: rest intervals) | - Productivity (a Likert scale of 5 point) | - Cardiorespiratory capacity (VO2max, 20-m shuttle run test, SRT) - Muscular strength (90° push-up test, standing long jump test) - BMI - Anxiety (a 4 point Likert scale) |  | | This pilot study provided preliminary evidence to support the feasibility and efficacy of the 8-week high-intensity interval training program on the variables analyzed. | |  | |  |
| (Michishita et al., 2017)^32^ | 130;  Intervention group (66)  45±11.2  Control group (64)  47±10.5 | 2 months | RCT | white- and blue-collar workers | To examine the effects of active rest by workplace units on vigor and presenteeism. | *Intervention group:* Active rest (short-time exercise) during lunch breaks  *Control group:*  No intervention | 10 minutes per day: three to four times per week for 8 weeks. The session include a warm-up (stretching), cognitive functional training (rotating movement of a pair of the same fingers with fixing other four pairs of fingers), aerobic exercise, body weight resistance training, and cool-down components. | - Abseenteism: the Work Functioning Impairment Scale (WFun) | - Amount of PA (steps/day) - BMI - Blood pressure |  | | The results suggest that the introduction of active rest program by workplace units provides a viable means for improving workplace vigor and presenteeism. | |  | |  |
| (de Vries et al., 2017)^33^ | 96;  Intervention group (49)  Control group (47)  F | 1 month and 2 weeks. | RCT | Participant from  healthcare organizations | To evaluated the efficacy of an exercise intervention to reduce work-related fatigue. | *Intervention group:*  Exercise intervention (EI). Running sessions  *Control group:* wait-list control group (WLC) | 1-hour low intensity, three times a week during 6 weeks. | - Workability (WAI) | - Cardiorespiratory capacity (VO2max, Urho Kaleva Kekkonen walk test) |  | | This study showed that exercise can serve as a relatively simple and inexpensive secondary prevention strategy to improve wellbeing among employees with high levels of work-related fatigue, especially if compliance is high. | |  | |  |
| (Brox & Froøystein, 2005)^34^ | 129;  Intervention group (63)  F(97%);  42.5  Control group (56)  F(96%) | 6 months | RCT | Nursing home | To evaluate the effectiveness of physical exercise at the workplace | *Intervention group*:  A weekly exercise class  *Control group:*  No intervention | 1-hour of exercise class consisting of light  aerobic exercise, muscle strengthening and stretching | - Absenteeism (self-reported questionnaire) | - Cardiorespiratory capacity (Aerobic fitness, The UKK walking test) - Health related PA (The COOP/WONCA charts) |  | | The intervention neither improved health-related quality of life nor reduced sickness absence. A modest improvement in aerobic capacity was observed. | |  | |  |
| (Edries et al., 2013)^35^ | 80  Intervention group (39)  Control group (41) | 6 months | RCT | clothing manufacturing workers | To evaluate the short-term effects of an employee wellness programme on clothing manufacturing employees. | *Intervention group:*  wellness programme based on the principles of cognitive behaviour therapy (CBT) as well as weekly supervised exercise classes over six weeks.  *Control group:*  A once-off health promotion talk and various educational pamphlets, with no further intervention | 30-minute health promotion talk and a 30-minute exercise class. The exercise class comprised of a brief warm-up, low to moderate intensity aerobics, core stability exercises and a cool down. The intensity and repetitions of the exercises were gradually increased each week. | - Absenteeism (number of days off/week from human resource manager) | - BMI - Anxiety: (EQ-5D questionnaire) |  | | The programme appeared to be beneficial in improving health related behaviours and absenteeism. | |  | |  |
| (Eriksen et al., 2002)^36^ | 860;  control (n= 344)  PE (n= 189), IHP (n= 165) SMT (n= 162) | 3 months | RCT | Post office workers | To evaluate the effect of 12 weeks of stress management training(SMT), physical exercise (PE) and an integrated health programme (IHP) on workers. | *Intervention group:*  SMT: Stress management training PE: Physical exercise  IHP: Integrated health programme  *Control group:*  No intervention | PE: a standardized aerobic dancing programme. The general aim was to improve physical capacity, muscle strength and flexibility.  IHP: physical exercise; information about stress, coping, health, nutrition, etc.; and practical examination at the worksite.  SMT: to improve the coping ability of the participants through a cognitive–behavioural approach. | - Absenteeism (last 30 days self-reported days off) | - Health related PA (subjective ad-hoc questionnaire) - Musculoskeletal symptoms (Health complaints inventory) - Stress (the Cooper Job Stress questionnaire) |  | | The PE group showed improved general health and muscle pain, while the SMT group showed improved stress management. The IHP group showed the strongest effects, affecting most goals set for treatment. | |  | |  |
