# Supplementary material for: Effectiveness of worksite wellness programs based on physical activity to improve workers’ health and productivity: a systematic review
Source: Syst Rev. 2023 May 24;12:87. doi: 10.1186/s13643-023-02258-6 (PMC10207792; doi:10.1186/s13643-023-02258-6)
Supplement: Supplementary file 3 — Additional file 3: Supplementary Table S3. Statistical parameters of the variables analyzed. [file 13643_2023_2258_MOESM3_ESM.docx]

| **Table S3:** Statistical parameters of the variables analyzed | | | | | | | | | | | | | | | | | | |  |  |  |  |  |
| --- | --- | --- | --- | --- | --- | --- | --- | --- | --- | --- | --- | --- | --- | --- | --- | --- | --- | --- | --- | --- | --- | --- | --- |
| Study | Outcomes | | | Intervention Group Control Group | | | | | | | | | | | | | | |  |  |  |  |  |
|  |  |  |  | Pre | Post | | Pre | | Post | | Effect size d | | | Power  (1-B err prob) | | | | |  |  |  |  |  |
| Santos et al. (2020)  Intervention group (98); Control group (106) | Musculoskeletal symptoms | | | 68.4 (12.1) | 59.4 (8.7) | | 56.2 (10.9) | | 51.7 (8.6) | 0.89 | | | | | 0.9999935 | | |  |  |  |  |  |  |
|  | Muscle Strength | | | 27.4 (2.4) | 29.7 (3.4) | | 27.7 (2.4) | | 30.3 (3.3) | 0.17 | | | | | 0.2463098 | | |  |  |  |  |  |  |
|  | Musculoskeletal symptoms | | | 30 (30.6) | 5 (5.1) | | 33 (33.7) | | 7 (6.6) | 0.34 | | | | | 0.6730552 | | |  |  |  |  |  |  |
|  | Physical activity level | | | 8.4 (0.8) | 9.5 (0.6) | | 8.4 (0.7) | | 9.5 (0.7) | 1.32 | | | | | 1.0000000 | | |  |  |  |  |  |  |
|  | Perceived risk | | | 58.2 (33.2) | 48.7 (25.7) | | 52.6 (35.2) | | 44.4 (28.5) | 0.16 | | | | | 0.2029957 | | |  |  |  |  |  |  |
|  | Physical fitness | | | 25 (4.2) | 24.6 (4.0) | | 25.3 (3.9) | | 24.8 (3.7) | 0.05 | | | | | 0.0657100 | | |  |  |  |  |  |  |
|  | Productivity | | | 7.2 (0.8) | 6.1 (0.7) | | 7.2 (0.8) | | 6.0 (0.7) | 1.26 | | | | | 1.0000000 | | |  |  |  |  |  |  |
| Lidegaard et al. (2018)  Intervention group (57);  Control Group (59) | Work ability | | | 9.23 | 9.60 (0.27) | | 9.21 | | 9.01 (0.27) | 2.18 | | | | | 1.0000000 | | |  |  |  |  |  |  |
|  | Productivity | | | 9.06 | 8.66 (0.33) | | 9.12 | | 8.57 (0.33) | 1.37 | | | | | 1.0000000 | | |  |  |  |  |  |  |
|  | Rating of perceived exertion | | | 7.83 | 7.98 (0.36) | | 8.00 | | 8.69 (0.36) | 1.97 | | | | | 1.0000000 | | |  |  |  |  |  |  |
| Hedwig et al.  (2020)  Intervention group (146);  Control Group (145) | Cardiorespiratory fitness | | | 25.4 ± 4.7 | 27.4 ± 5.2 | | 25.7 ± 5.1 | | 25.7 ± 5.1 | 0.33 | | | | | 0.42 | | |  |  |  |  |  |  |
|  | body weight (kg) | | | 72.3 ± 13.9 | 71.6 ± 13.5 | | 72.6 ± 13.6 | | 72.6 ± 13.3 | 0.07 | | | | | 0.0975616 | | |  |  |  |  |  |  |
|  | Body fat percentage | | | 32.9 ± 7.3 | 32.2 ± 7.5 | | 33.2 ± 7.8 | | 33.8 ± 7.4 | 0.21 | | | | | 0.4491534 | | |  |  |  |  |  |  |
|  | Amount of Physical Activity | | | 4.8 ± 4.9 | 14.3 ± 11.4 | | 5.1 ± 5.2 | | 8.5 ± 15.3 | 0.43 | | | | | 0.9561033 | | |  |  |  |  |  |  |
|  | Work Ability Index | | | 7.8 ± 1.3 | 8.2 ± 1.2 | | 8.2 ± 1.3 | | 8.1 ± 1.3 | 0.08 | | | | | 0.1047333 | | |  |  |  |  |  |  |
| Song and Baicker (2020)  Intervention group (4037)  Control group (28937) | Stress | | |  | 56.2 (49.6) | |  | | 55.7 (49.7) | 0.01 | | | | | 0.0920674 | | |  |  |  |  |  |  |
|  |  |  |  |  |  |  |  |  |  |  |  |  |  |  |  |  |  |  |  |  |  |  |  |
|  | Regular exercise | | |  | 69.8(46.0) | |  | | 61.9 (48.6) | 0.17 | | | | | 1.0000000 | | |  |  |  |  |  |  |
|  | Cholesterol | | |  | 180.9 (44.4) | |  | | 177.6 ( 41.5) | 0.07 | | | | | 0.9954796 | | |  |  |  |  |  |  |
|  | BMI | | |  | 29.9 (7.1) | |  | | 29.7 (7.1) | 0.03 | | | | | 0.02 | | |  |  |  |  |  |  |
|  | Hypertension | | |  | 26.5 (44.2) | |  | | 23.1 (42.2) | 0.08 | | | | | 0.9967670 | | |  |  |  |  |  |  |
|  | Absenteeism | | | \|  \| 2.6 (1.6) \| \| --- \| --- \| | 2.5 (1.6) | |  | | 2.6 (1.6) | 0.06 | | | | | 0.9607953 | | |  |  |  |  |  |  |
|  | Productivity | | | \|  \| 60.5 (48.9) \| \| --- \| --- \| | 60.6 (48.9) | |  | | 60.5 (48.9) | 0.00 | | | | | 0.0516988 | | |  |  |  |  |  |  |
| Hartfiel et al.  (2017)  Intervention group (76)  Control group (75) | Musculoskeletal symptoms | | | 2.09 (2.44) | 1.34 (1.72) | | 1.93 (2.97) | | 2.36 (3.44) | 0.24 | | | | | 0.3173552 | | |  |  |  |  |  |  |
|  | Anxiety | | | 1.37 (1.16) | 0.76 (0.77) | | 1.41 (1.40) | | 1.62 (1.36) | 0.58 | | | | | 0.9396600 | | |  |  |  |  |  |  |
|  | Abseenteism | | | 0.84 (0.02) | 0.86 (0.02) | | 0.82 (0.02) | | 0.78 (0.02) | 1.00 | | | | | 0.9999829 | | |  |  |  |  |  |  |
| Pedersen et al. (2009)  SRT group (180)  APE group (187)  REF group  (182)  Justesenet al. (2017)  Intervention group (N= 193) Control group (N= 194) | Physical Activity | | |  |  | |  | |  |  | | | | |  | | |  |  |  |  |  |  |
|  | Physical Capacity. | | |  |  | |  | |  |  | | | | |  | | |  |  |  |  |  |  |
|  | Musculoskeletal symptoms. | | |  |  | |  | |  |  | | | | |  | | |  |  |  |  |  |  |
|  | BMI | | |  |  | |  | |  |  | | | | |  | | |  |  |  |  |  |  |
|  | General Health | | |  |  | |  | |  |  | | | | |  | | |  |  |  |  |  |  |
|  | Productivity | | |  |  | |  | |  |  | | | | |  | | |  |  |  |  |  |  |
|  | Workability | | | 8.7 (1.2) | 8.8 (1.1) | | 8.8 (0.9) | 8.9 (1.0) | | | | 0.20 | | | | 0.4970990 | | | | | |  |  |
|  | Productivity | | | 8.3 (1.1) | 8.6 (1.1) | | 8.2 (1.3) | | 8.3 (1.0) | 0.29 | | | | | 0.7995938 | | |  |  |  |  |  |  |
|  | Self-rated health | | | 3.6 (0.7) | 3.8 (0.7) | | 3.7 (0.8) | | 3.6 (0.6) | 0.15 | | | | | 0.3248434 | | |  |  |  |  |  |  |
|  | Sickness absence | | | 4.4 (6.3) | 3.2 (5.3) | | 3.5 (4.7) | | 3.4 (4.4) | 0.04 | | | | | 0.0687922 | | |  |  |  |  |  |  |
| Dalager et al. (2016)  Intervention group (N= 193) Control group (N= 194)  Dalager et al.  (2015)  WS Intervention group= 116; 3WS Intervention group= 126; 9WS Intervention group= 106; 3MS Intervention group= 124; Control group= 101 | BMI | | | 25.3 (5.0) 25.08 (1.09) | | | 25.5 (5.2) 25.56 (1.06) | | | 0.45 | | | | | 0.9922520 | | |  |  |  |  |  |  |
|  | Relative VO2max | | | 36.3 (11.3) 37.76 (6.57) | | | 35.8 (11.1) 35.98 (6.40) | | | 0.27 | | | | | 0.7681858 | | |  |  |  |  |  |  |
|  |  | | |  | | |  | | |  | | | | |  | | |  |  |  |  |  |  |
|  |  | | | | |  |  |  |  |  |  |  |  |  |  |  |  |  |  |  |  |  |  |
|  |  |  |  |  |  |  |  |  |  |  |  |  |  |  |  |  |  |  |  |  |  |  |  |
|  | Workability (scale 0-10) | | | 1 WS= 9.7 (1.3)  3 WS= 9.9 (1.1)  9WS= 9.9 (1.1)  3MS=  9.9 (1.2) | 1 WS=  9.8 (1.0)  3 WS=  9.9 (1.0)  9WS= 10.1 (1.0)  3MS=  9.8 (1.4) | | 9.9 (1.1) | | 9.9 (1.0) | 0.1  0  0.20  0.08 | | | | | 0.1807844  0.0500000  0.2989688  0.0936910 | | |  |  |  |  |  |  |
|  | Productivity (scale 0-10) | | | 1 WS= 9.2 (1.3)  3 WS= 9.3 (1.1)  9WS= 9.4 (1.1)  3MS=  9.1 (1.4) | 1 WS=  9.2 (1.2)  3 WS=  9.9 (1.1)  9WS= 9.5 (1.1)  3MS=  9.1 (1.4) | | 9.1 (1.4) | | 9.2 (1.3) | 0  0.60  0.25  0.08 | | | | | 0.0500000  0.9944567  0.4299795  0.0981683 | | |  |  |  |  |  |  |
|  | Muscular strength | | | 1 WS= 6.8 (2.6)  3 WS=7.5 (2.8)  9WS=  6.7 (3.0)  3MS=  7.9 (2.6) | 1 WS=  7.6 (2.8)  3 WS=  8.0 (3.1)  9WS=  7.0 (3.0)  3MS=  8.6 (2.8) | | 7.8 (2.7) | | 8.0 (2.8) | 0.14  0  0.34  0.21 | | | | | 0.2747652  0.0500000  0.6938632  0.3565816 | | |  |  |  |  |  |  |
|  |  |  |  |  |  |  |  |  |  |  |  |  |  |  |  |  |  |  |  |  |  |  |  |
| Puig-Ribera, et al.  (2008)  Intervention group Walking Routes (WR)= n= 26; Intervention group Walking in task (WT) = 26 Control group (N= 27) | Step counts | | | WR= 9662 (2855)  WT= 9216 (2921) | WR= 9748 (2543)  WT= 9228 (2426) | | 9855 (3664) | | 9664 (1620) | 0.04  0.21 | | | | | 0.0522711  0.1173954 | | |  |  |  |  |  |  |
|  |  |  |  |  |  |  |  |  |  |  |  |  |  |  |  |  |  |  |  |  |  |  |  |
|  |  |  |  |  |  |  |  |  |  |  |  |  |  |  |  |  |  |  |  |  |  |  |  |
| Eather et al.  (2020)  Intervention group (N= 24) Control group (N= 23) | BMI | | | 24.34 (4.00) | 24.77 (3.44) | | 25.72 (4.07) | | 25.87 (4.30) | 0.28 | | | | | 0.2447052 | | |  |  |  |  |  |  |
|  | Muscular strength | | | 126.33 (37.57) | 135.88 (29.51) | | 134.5 (31.55) | | 133.91 (27.55) | 0.07 | | | | | 0.0561613 | | |  |  |  |  |  |  |
|  | Cardiorespiratory fitness | | | 26.00 (20.14) | 34.71 (21.40) | | 33.50 (19.35 | | 39.24(21.75) | 0.02 | | | | | 0.1084739 | | |  |  |  |  |  |  |
|  | Anxiety | | | 6.04 (5.33) | 5.16 (3.30) | | 5.14 (4.76) | | 4.11 (3.81) | 0.29 | | | | | 0.1671762 | | |  |  |  |  |  |  |
|  | Productivity | | | 3.87 (0.52) | 4.15 (0.55) | | 3.78 (0.61) | | 3.79 (0.60) | 0.63 | | | | | 0.5548120 | | |  |  |  |  |  |  |
| Michishita et al.  (2017)  Intervention group (66);  Control group (64)  de Vries et al. (2017)  Intervention group (49)  Control group (47) | Physical activity levels (steps/day) | | | 9062 (3556) | 10525 (4684) | | 9681 (3760) | | 10792 (4404) | 0.06 | | | | | 0.0627412 | | |  |  |  |  |  |  |
|  | Anthropometric indices and blood pressure | | | 24.2 (4.1) | 24.0 (3.9) | | 23.6 (3.9) | | 23.5 (3.9) | 0.13 | | | | | 0.1121057 | | |  |  |  |  |  |  |
|  | Abseenteism (WFun) | | | 14.4 (6.4) | 12.6 (5.5) | | 16.5 (6.4) | | 17.2 (6.8) | 0.74 | | | | | 0.9877110 | | |  |  |  |  |  |  |
|  | Work ability (1–10) | | | 7.73 (1.32) | 7.96 (1.58) | | 7.08 (2.20) | | 6.90 (2.14) | 0.56 | | | | | 0.7799206 | | |  |  |  |  |  |  |
|  | VO2max | | | 30.89 (6.02) | 32.18 (7.10) | | 27.65 (6.01) | | 29.72 (7.07) | 0.34 | | | | | 0.3911304 | | |  |  |  |  |  |  |
| Brox et al. (2005)  Intervention group (63);  Control group (56) | Mean UKK fitness test score | | | 86.2 (15.6) | 94.4 (12.4) | | 85.2 (12.4) | | 90.9 (9.6) | 0.39 | | | | | 0.5725492 | | |  |  |  |  |  |  |
|  | Sickness absence (mean days) | | | 6.8 (14.6) | 15.6 (28.4) | | 10.4 (22.0) | | 14.4 (22.9) | 0.05 | | | | | 0.0572601 | | |  |  |  |  |  |  |
|  | Overall health | | | 2.2 (0.7) | 2.2 (0.9) | | 2.1 (0.6) | | 2.1 (0.7) | 0.12 | | | | | 0.1027737 | | |  |  |  |  |  |  |
| Edries et al. (2013)  Intervention group (39)  Control group (41) | BMI | | | 28.90 (6.29) |  | | 29.80 (9.37) | |  |  | | | | |  | | |  |  |  |  |  |  |
|  | Anxiety/Depression | | | 8 | 10 | | 12 | | 14 |  | | | | |  | | |  |  |  |  |  |  |
| Eriksnn  (2002)  Intervention group PE (189); IHP (165); SMT (162)  Control group (344) | Abseentism | | | PE: 1.38 (0.80–1.96)  IHP: 2.27 (0.95–3.59)  SMT: 1.41 (0.72–2.10) | PE: 0.65 (0.21–1.10)  IHP: 1.43 (0.72–2.15)  SMT: 1.27 (0.55–2.00) | | 1.51 (0.99–2.03) | | 1.60 (0.97–2.22) |  | | |  | | | |  | | | | | |  |
|  | Job stress | | | PE: 11.25 (9.71–12.78)  IHP: 10.78 (9.03–12.53)  SMT: 12.73 (10.95–14.51) | PE: 9.77 (8.55–11.00)  IHP: 9.77 (8.37–11.17)  SMT:  2.51 (11.08–13.95) | | 11.54 (10.16–12.92) | | 11.78 (10.69-12.88) |  | | |  | | | |  | | | | | |  |
|  |  |  |  |  |  |  |  |  |  |  |  |  |  |  |  |  |  |  |  |  |  |  |  |
